# Association and interaction of O_3_ and NO_2_ with emergency room visits for respiratory diseases in Beijing, China: a time-series study

**DOI:** 10.1186/s12889-022-14473-2

**Published:** 2022-12-05

**Authors:** Yuanwei Fu, Wenlou Zhang, Yan Li, Hongyu Li, Furong Deng, Qingbian Ma

**Affiliations:** 1 Emergency Department, Peking University Third Hospital, Beijing, 100191 China; 2 Department of Occupational and Environmental Health Sciences, School of Public Health, Peking University, Beijing, 100191 China

**Keywords:** Air pollution, Ozone, Nitrogen dioxide, Emergency room visits, Respiratory diseases, Interaction

## Abstract

**Background:**

Ozone (O_3_) and nitrogen dioxide (NO_2_) are the two main gaseous pollutants in the atmosphere that act as oxidants. Their short-term effects and interaction on emergency room visits (ERVs) for respiratory diseases remain unclear.

**Methods:**

We conducted a time-series study based on 144,326 ERVs for respiratory diseases of Peking University Third Hospital from 2014 to 2019 in Beijing, China. Generalized additive models with quasi-Poisson regression were performed to analyze the association of O_3_, NO_2_ and their composite indicators (O_x_ and O_x_^wt^) with ERVs for respiratory diseases. An interaction model was further performed to evaluate the interaction between O_3_ and NO_2_.

**Results:**

Exposure to O_3_, NO_2_, O_x_ and O_x_^wt^ was positively associated with ERVs for total respiratory diseases and acute upper respiratory infection (AURI). For instance, a 10 μg/m^3^ increase in O_3_ and NO_2_ were associated with 0.93% (95%CI: 0.05%, 1.81%) and 5.87% (95%CI: 3.92%, 7.85%) increase in AURI at lag0-5 days, respectively. Significant linear exposure–response relationships were observed in O_x_ and O_x_^wt^ over the entire concentration range. In stratification analysis, stronger associations were observed in the group aged < 18 years for both O_3_ and NO_2_, in the warm season for O_3_, but in the cold season for NO_2_. In interaction analysis, the effect of O_3_ on total respiratory emergency room visits and AURI visits was the strongest at high levels (> 75% quantile) of NO_2_ in the < 18 years group.

**Conclusions:**

Short-term exposure to O_3_ and NO_2_ was positively associated with ERVs for respiratory diseases, particularly in younger people (< 18 years). This study for the first time demonstrated the synergistic effect of O_3_ and NO_2_ on respiratory ERVs, and O_x_ and O_x_^wt^ may be potential proxies.

**Supplementary Information:**

The online version contains supplementary material available at 10.1186/s12889-022-14473-2.

## Introduction

In China, respiratory emergencies are one of the major pre-hospital emergency medical service (EMS) demand and show an increasing trend in recent years [1]. Respiratory diseases are one of the leading causes of both morbidity and mortality worldwide, seriously threatening global health. Pneumonia, chronic obstructive pulmonary disease (COPD) and asthma are main contributors of both respiratory-related mortality and morbidity [2].Recent studies have shown positive associations between short-term exposure to air pollution and emergency room visits (ERVs) for respiratory diseases and cause-specific mortality [3–6].

Ozone (O_3_) pollution in China has been on the rise in recent years, and in most regions of the world, it is also not optimistic; Nitrogen dioxide (NO_2_) is a traffic-related pollutant with high levels in most parts of the world because of increasing traffic, and its production is closely related to O_3_ [7, 8]. Previous studies revealed that air pollutants had different effects on ERVs for respiratory diseases and they may pose a combined effect [9]. Particulate matter showed a dominant effect on respiratory visits, however, few studies have focused on the short-term effects of O_3_ and NO_2_ on ERVs for respiratory diseases, and the results varied by city, age and sex [9, 10].

Both O_3_ and NO_2_ are major gaseous pollutants with strong oxidative ability. The health effects of O_3_ and NO_2_ may not be independent, because of their common oxidative properties that can lead to oxidative stress, as well as the inextricably chemical conjunction that result from their rapid reactions in the atmosphere [8]. Therefore, there is increasing interest in using the sum of O_3_ and NO_2_ (O_x_) as an indicator of the combined oxidant capacity [8] [11, 12]. In addition, it is well known that the oxidation potential of O_3_ is much stronger than NO_2_, so the term ‘redox-weighted oxidant capacity’ (O_x_^wt^) is derived as a weighted average using redox potentials as the weights [11]. Several previous studies have been conducted on the relationship between air pollutants and disease morbidity and mortality, in which the combined atmospheric oxidant capacity is represented by the redox-weighted average of O_3_ and NO_2_ [12–16]. However, evidence regarding the effects of O_x_ and O_x_^wt^ on respiratory emergency visits is lacking.

In real-world scenarios, people are always exposed to a range of harmful air pollutants simultaneously. It is biologically plausible for the potential interaction of different pollutants on human health. For example, a case-crossover study conducted in Canada found that the association between fine particulate matter (PM_2.5_) and emergency room visits for myocardial infarction was stronger in areas with higher O_x_^wt^ (*P*-interaction < 0.001) [12]. Another time-series analysis observed a positive interaction between inhalable particles (PM_10_) and NO_2_ on non-accidental mortality in Guangzhou, China [17]. Furthermore, a panel study found that the effect of O_3_ on cardiac autonomic function were stronger at high levels of black carbon (BC), another surrogate indicator of traffic emissions, in children in Beijing, China [18]. It is important for assessing the overall health risk of air pollution to understand these possible interactions. But so far, the interaction between O_3_ and NO_2_ has not been investigated. To address these gaps, this time-series study was conducted to estimate the association of short-term exposure to O_3_, NO_2_ and their combined indicators (O_x_ and O_x_^wt^) with ERVs for respiratory diseases and explore the interaction of O_3_ and NO_2_.

## Materials and methods

### Study design and population

We conducted a time-series study based on the emergency room visits data of Peking University Third Hospital (PUTH) from Jan 1, 2014 to Dec 31, 2019. For more than ten years, the number of outpatients and emergency visits of PUTH has always been in the forefront in Beijing. In 2019, the hospital served more than 4.22 million outpatients and over 300,000 emergency patients with different kinds of demographical characteristics. The data of daily hospital ERVs for respiratory diseases were collected from the hospital information system from January 2014 to December 2019. The cases were classified according to the 10th edition of the International Classification of Disease (ICD-10): (1) total respiratory diseases (TRDs, J00-J99); (2) acute upper respiratory infection (AURI, J00-J06); (3) lower respiratory tract infection (LRTI, J12-J18&J20-J22); (4) pneumonia (J12-J18); (5) chronic obstructive pulmonary disease (COPD, J41-J44); and (6) asthma (J45).

### Air pollutants and meteorological data

Hourly concentrations of six major ambient air pollutants were obtained from the Chinese Air Quality Online Monitoring and Analysis Platform (https://www.aqistudy.cn/). There were 35 air quality monitoring stations in Beijing, which could well represent the level of air pollution in Beijing. Daily average concentrations of PM_2.5_, PM_10_, NO_2_, sulfur dioxide (SO_2_), carbon monoxide (CO) and 8-h maximum concentration of O_3_ (O_3_-8 h max) were calculated during the study period. Meanwhile, daily meteorological data were collected from the National Meteorological Information Center (http://data.cma.cn/), including temperature and relative humidity (RH) in Beijing. In addition, O_3_ and NO_2_ are the two main oxidative atmospheric pollutants, and there is a mutual chemical transformation between them. This study calculated two composite indicators (O_x_ and O_x_^wt^) based on previous studies. O_x_ was defined as the sum of NO_2_ and O_3_ concentrations indicating combined oxidant capacity [8]. O_x_^wt^ was defined as the weighted average of NO_2_ and O_3_ indicating redox-weighted oxidant capacity because O_3_ is more oxidizing than NO_2_ [8]. The calculation formula is as follows:

1 $$O_x=O_3+NO_2$$

2 $$O_x^{\mathrm{wt}}=(1.07volts(\mathrm V)\times\;{\mathrm{NO}}_2+\;2.075\mathrm{volts}(\mathrm V)\times\;{\mathrm O}_3)/3.145$$

### Statistical analysis

Generalized additive models with quasi-Poisson regression were performed to analyze the association between air pollutants and ERVs for respiratory diseases according to the formula (3):

3 $$\mathrm{Log}\lbrack\mathrm E({\mathrm Y}_{\mathrm t})\rbrack\;=\;{\mathrm\beta}_0\;+\;{\mathrm\beta}_1{\mathrm Z}_{\mathrm t}\;+\;\mathrm{Cb}.\mathrm{temp}\;+\;\mathrm{Day}\;\mathrm{of}\;\mathrm{Week}\;+\;\mathrm{Holiday}\;+\;\mathrm{ns}(\mathrm{time},\;7\;\mathrm{df}\;\times\;\mathrm{year})\;+\;\mathrm{ns}(\mathrm{RH},\;3\;\mathrm{df})$$

where *t* is the day of the observation; *E*(*Y*_*t*_) is the expected value of ERVs for respiratory diseases on day *t*; β_0_ is the intercept; β_1_ is the regression coefficient of exposure; *Z*_*t*_ is the moving average concentration of pollutants at different lag days; Cb.temp is a cross-basis matrix of temperature generated by the distributed lag nonlinear model (DLNM) with 4 df and a maximum lag days of 14; Day of Week (DOW) and Holiday variables are used to adjust the short-term variation; ns() is a natural cubic spline function; time is the calendar time on day *t*; RH is the relative humidity. The dfs for the temperature, time and RH was determined based on the common dfs used in previous studies [19–22]. Then, we plotted the exposure–response curves to characterize the associations between air pollutants and daily ERVs for respiratory diseases at different exposure concentrations [23]. We evaluated the lag effects for a maximum of 0–7 days after exposure to air pollutants and found the strongest cumulative effects at lag 0–5 days, which were finally reported.

We performed additional analyses stratified by sex (male and female), age (< 18, 18–64 and > 64 years) and season (warm: May to October and cold: November to April) with reference to previous studies [24, 25]. The formula (4) was used to test the statistical differences between different groups by calculating the 95% confidence interval (CI), which was used widely in previous studies [13, 23, 26]. For age stratification analysis, 18–64 years group was used as a reference.

4 $$Q_{1}-Q_{2}\pm1.96\sqrt{{\left(SE_{1}\right)}^{2}+{\left(SE_{2}\right)}^{2}}$$

For interaction analysis, NO_2_ or O_3_ concentrations were classified as low, medium, and high levels according to the 25% and 75% quartiles based on previous studies [17, 19], and then the associations between one pollutant and ERVs for respiratory diseases under different levels of the other pollutant were explored based on the formula (5) [17, 19]:

5 $$\mathrm{Log}\lbrack\mathrm E({\mathrm Y}_{\mathrm t})\rbrack\;=\;{\mathrm\beta}_{0}\;+\;{\mathrm\beta}_{1}{\mathrm Z}_{1}\;+\;{\mathrm\beta}_{2}({\mathrm Z}_{1}:{\mathrm Z}_{2})+\;\mathrm{Cb}.\mathrm{temp}\;+\;\mathrm{DOW}\;+\;\mathrm{Holiday}\;+\;\mathrm{ns}(\mathrm{time},\;7\;\mathrm{df}\;\times\;\mathrm{year})\;+\;\mathrm{ns}(\mathrm{RH},\;3\;\mathrm{df})$$

where *Z*_*1*_ and *Z*_*2*_ are O_3_ and NO_2_ levels (or NO_2_ and O_3_ levels), respectively; β_2_ is the effect of the interaction between *Z*_*1*_ and *Z*_*2*_. Other parameters are the same as in formula (3). The low concentration group of pollutants was used as the reference, so the estimated effect of O_3_ at a low NO_2_ level (or NO_2_ at a low O_3_ level) was the same as the β_1_ of formula (5), and the estimated effects of one pollutant at medium and high levels of another pollutant were generated based on both β_1_ and β_2_.

Sensitivity analyses were also performed to check the robustness of the results by: (1) constructing two-pollutant models by including PM_2.5,_ PM_10_, SO_2_ or CO; (2) modifying the maximum lag time for temperature from 14 to 30 days [19, 27]; and (3) modifying the degree of freedom for calendar time from 4 to 10 [28–30]. We calculated the variance inflation factor (VIF) for the two-pollutant models and found the corresponding VIFs were all less than 5, indicating that the collinearity was not an issue for the two-pollutant models.

All statistical analyses were performed using R software (Version 4.0.3) with “mgcv” and “dlnm” packages. A two-sided *P* < 0.05 was considered statistically significant.

## Results

### Descriptive statistics

A total of 144,326 emergency room visits for respiratory diseases were included in this study from 2014–2019, among which 80,302 (55.6%) were acute upper respiratory infection cases, and 24,621 (17.1%) were lower respiratory tract infection cases (Table 1). During the study period (2014–2019), the average daily emergency room visits were 65.9 (range: 12 to 444). Pneumonia was the most common cause of lower respiratory tract infection. COPD and asthma cases were 2553 (1.8%) and 2740 (1.9%), respectively (Table 1). The daily mean (standard deviation, SD) concentrations of 8 h maximum O_3_ and NO_2_ were 98.1 (62.8) and 45.5 (22.5) μg/m^3^, respectively. The mean (SD) of temperature and relative humidity were 13.9 (11.2)°C and 50.8(19.8)%, respectively (Table 2). The daily concentration changes of air pollutants were shown in Figure S1. The O_3_ had high concentrations in warm season and in contrast, NO_2_ had high concentrations in cold season. According to the results of Spearman correlation analysis, O_3_ was negatively correlated with NO_2_ (coefficient = -0.35, *P* < 0.001, Figure S2A) throughout the study period, and this correlation was stronger in the cold season (coefficient = -0.45, *P* < 0.001, Figure S2C).

**Table 1:** Descriptive statistics of emergency room visits for respiratory diseases from Jan 1, 2014 to Dec 31, 2019

Variable	TRD	AURI	LRTI	Pneumonia	COPD	Asthma
N	144,326	80,302	24,621	24,484	2,553	2,740
Sex, n(%)						
Male	74,489 (51.6%)	38,834 (48.4%)	13,124 (53.3%)	13,060 (53.3%)	1,622 (63.5%)	1,181 (43.1%)
Female	69,837 (48.4%)	41,468 (51.6%)	11,497 (46.7%)	11,424 (46.7%)	931 (36.5%)	1,559 (56.9%)
Age, n(%)						
< 18 years	48,937 (33.9%)	41,134 (51.2%)	4,237 (17.2%)	4,234 (17.3%)	0 (0.0%)	276 (10.1%)
18–64 years	65,099 (45.1%)	36,080 (44.9%)	8,856 (36.0%)	8,750 (35.7%)	314 (12.3%)	1,776 (64.8%)
> 64 years	30,290 (21.0%)	3,088 (3.8%)	11,528 (46.8%)	11,500 (47.0%)	2,239 (87.7%)	688 (25.1%)

*TRD* Total respiratory disease, *AURI* Acute upper respiratory infection, *LRTI* Lower respiratory tract infection, *COPD* Chronic obstructive pulmonary disease

**Table 2:** Descriptive statistics for air pollutants and meteorological conditions from Jan 1, 2014 to Dec 31, 2019 in Beijing

Variable	Mean ± SD	Median	IQR
O_3_-8 h max, μg/m^3^	98.1 ± 62.8	83.0	88.0
NO_2_, μg/m^3^	45.5 ± 22.5	40.0	26.8
O_x_, μg/m^3^	143.7 ± 58.9	125.0	85.0
O_x_^wt^, μg/m^3^	80.2 ± 39.4	67.5	57.6
PM_2.5_, μg/m^3^	64.2 ± 59.4	47.0	60.0
PM_10_, μg/m^3^	91.5 ± 66.7	75.0	73.0
SO_2_, μg/m^3^	10.2 ± 13.6	5.0	8.0
CO, mg/m^3^	1.0 ± 0.8	0.8	0.6
T, ℃	13.9 ± 11.2	15.3	21.5
RH, %	50.8 ± 19.8	51.0	31.5

*PM*_*2.5*_ Fine particles, *PM*_*10*_ Inhalable particles, *NO*_*2*_ Nitrogen dioxide, *O*_*3*_ Ozone, *SO*_*2*_ Sulfur dioxide, *CO* Carbon monoxide, *O*_*X*_ Oxidant capacity, *O*_*x*_^*wt*^ Redox-weighted oxidant capacity, *T* Temperature, *RH* Relative humidity, *SD* Standard deviation, *IQR* Interquartile range

### Associations between ambient air pollutants and ERVs for respiratory diseases

**Fig. 1:**
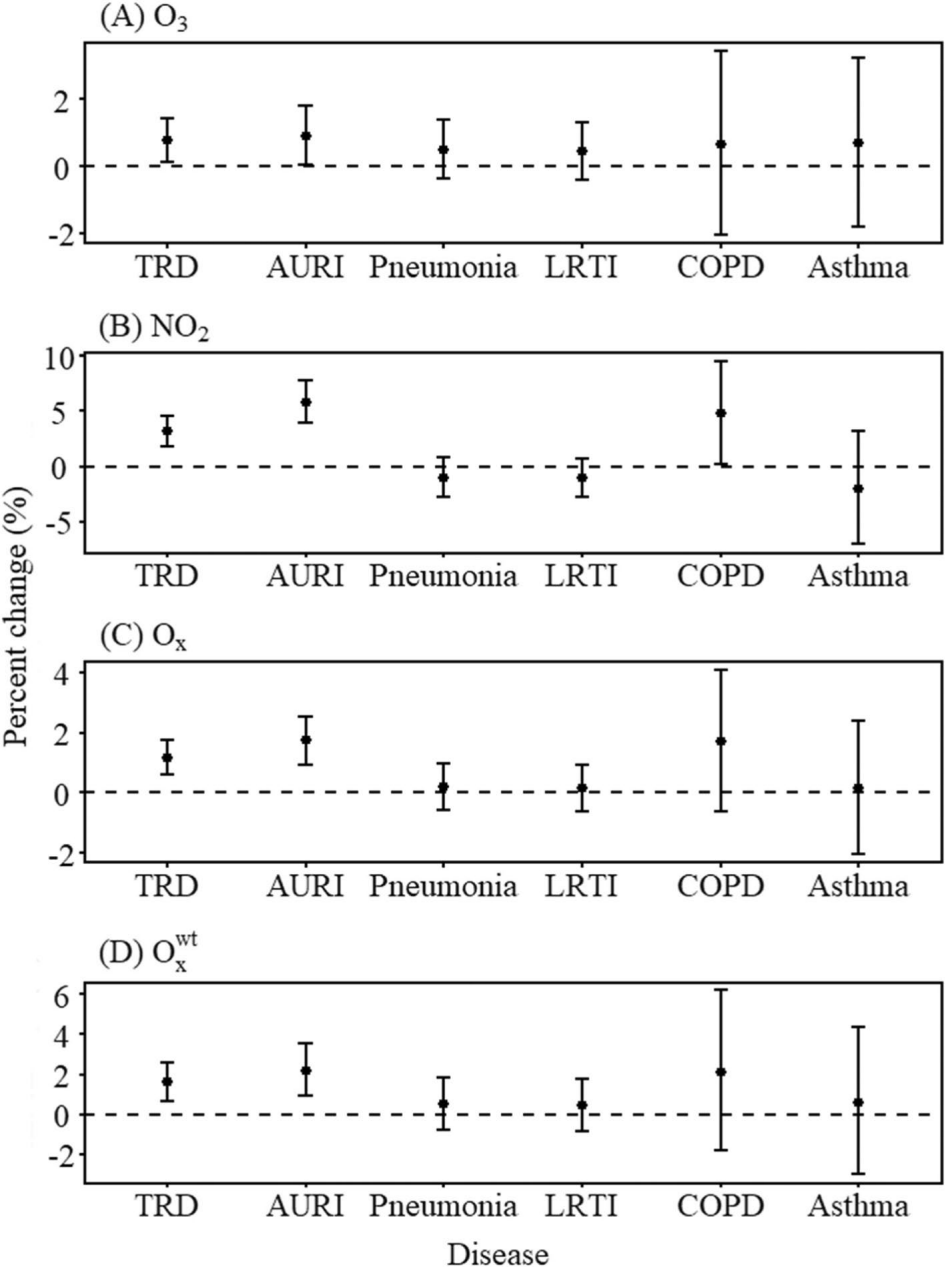
Associations of air pollutants (O_3_, NO_2_, O_X_ and O_x_^wt^) with emergency room visits for respiratory diseases at lag 05 day during 2014–2019. NOTE: O_3_, ozone; NO_2_, nitrogen dioxide; O_X_, oxidant capacity; O_x_^wt^, redox-weighted oxidant capacity; TRD, total respiratory disease; AURI, acute upper respiratory infection; LRTI, lower respiratory tract infection; COPD, chronic obstructive pulmonary disease

As shown in Fig. 1, short-term exposure to O_3_, NO_2_, O_x_ and O_x_^wt^ was positively associated with emergency room visits for total respiratory diseases and acute upper respiratory infection. For instance, a 10 μg/m^3^ increase in O_3_ and NO_2_ were associated with 0.78% (95%CI: 0.14%, 1.42%) and 3.17% (95%CI: 1.82%, 4.53%) increase in total respiratory visits and 0.93% (95%CI: 0.05%, 1.81%) and 5.87% (95%CI: 3.92%, 7.85%) increase in acute upper respiratory infection, respectively. A positive correlation was observed between NO_2_ and COPD but not with asthma. However, NO_2_, O_3_, O_x_ and O_x_^wt^ were not significantly associated with lower respiratory tract infection. Figure S3 showed the exposure–response relationship between air pollutants and ERVs for respiratory diseases. O_3_ showed a positive correlation with total respiratory visits and acute upper respiratory infection at concentrations above 100 μg/m^3^, and NO_2_ showed a positive correlation with acute upper respiratory infection. O_x_ and O_x_^wt^, on the other hand, showed positive correlations with total respiratory visits and acute upper respiratory infection over the entire concentration range, suggesting that they may be a better indicator for assessing health effects following mixed O_3_ and NO_2_ exposure.

### Stratification analysis

**Fig. 2:**
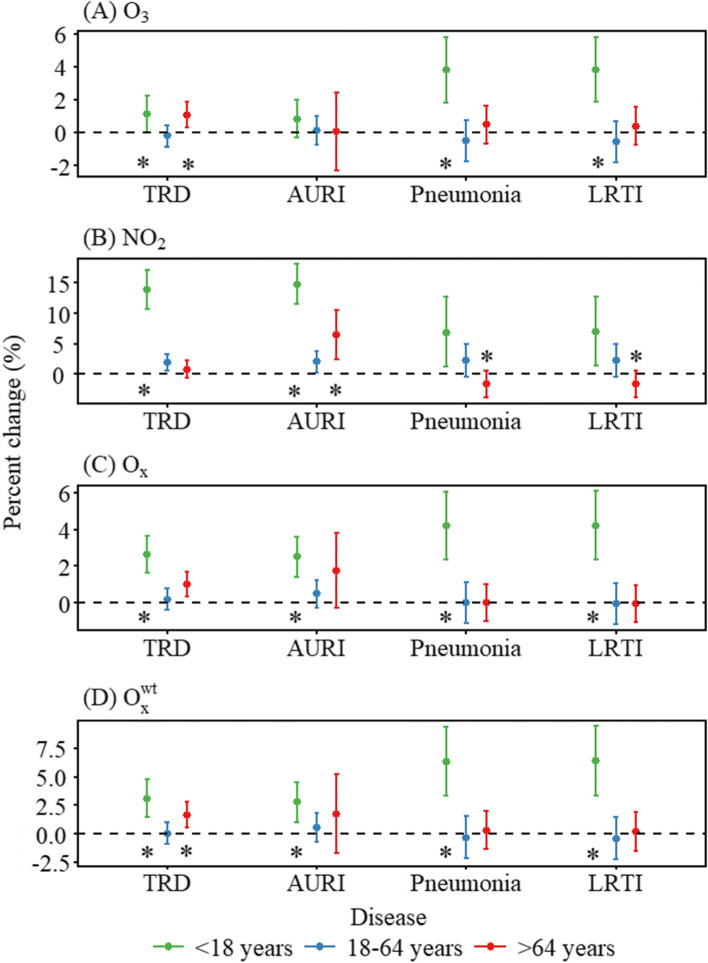
Associations of air pollutants (O_3_, NO_2_, O_X_ and O_x_^wt^) with emergency room visits for respiratory diseases stratified by age at lag 05 day during 2014–2019. NOTE: O_3_, ozone; NO_2_, nitrogen dioxide; O_X_, oxidant capacity; O_x_^wt^, redox-weighted oxidant capacity; TRD, total respiratory disease; AURI, acute upper respiratory infection; LRTI, lower respiratory tract infection.^*^P for subgroup differences < 0.05 compared with the group aged 18–64 years

Gender-stratified associations between air pollutants and respiratory emergency room visits were shown in Figure S4. Stronger associations were observed in females, although statistically significant gender differences were found only in the association between NO_2_ and lower respiratory tract infection. The results of age-stratified associations are shown in Fig. 2. We found the strongest association in the group aged < 18 years and almost all the differences were statistically significant (*P* < 0.05) compared with the group aged 18–64 years. Certain associations were also observed to be stronger in the group aged > 64 years than in the group aged 18–64 years, such as the association of NO_2_ with acute upper respiratory infection. Figure S5 showed the results of season-stratified analysis. Overall, the effects of 8 h maximum O_3_ were significantly greater in the warm season (all *P* < 0.05), while the effect of NO_2_ was slightly stronger in the cold season, and the difference was statistically significant only for the acute upper respiratory infection.

### Interaction analysis

**Fig. 3:**
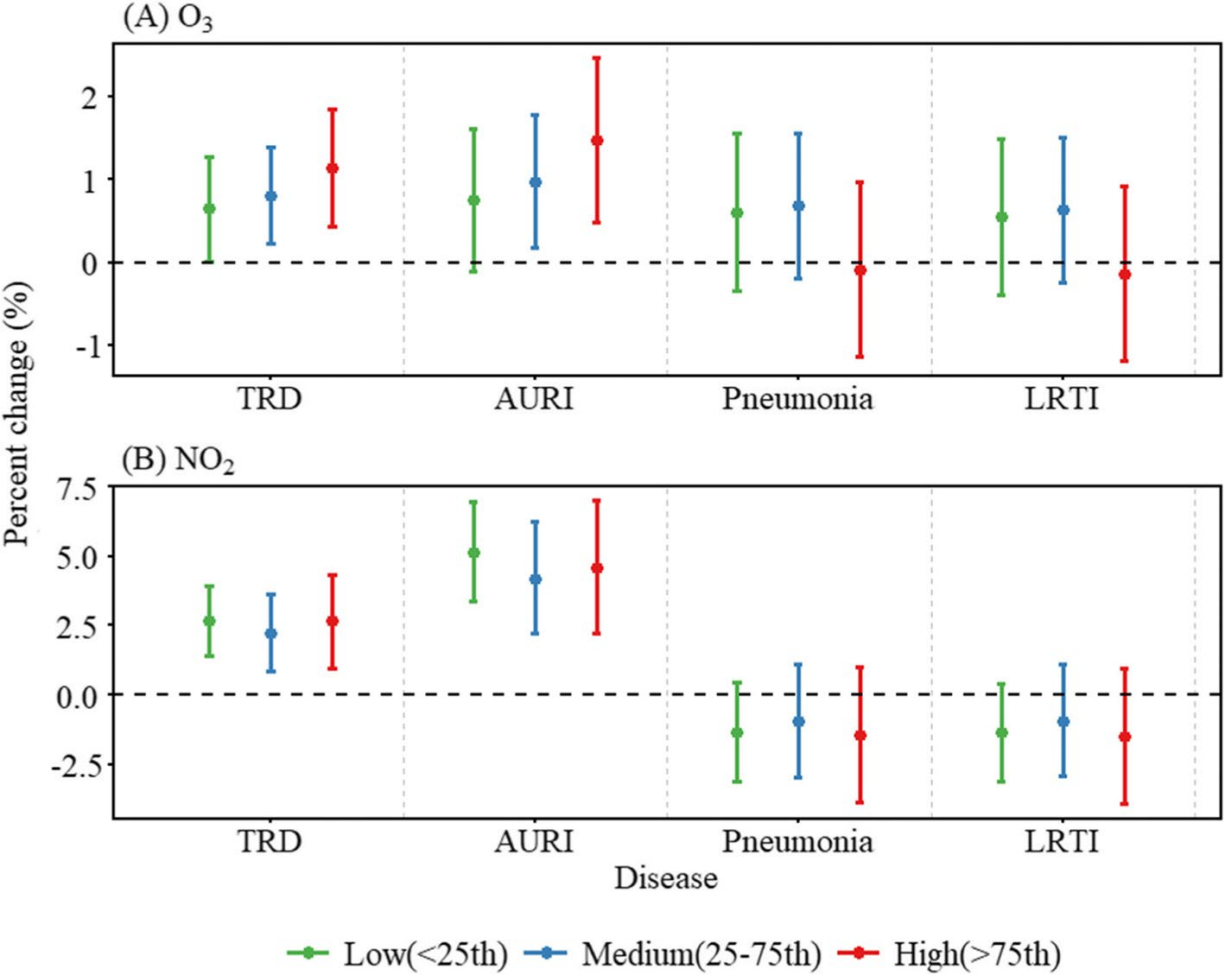
The interaction of O_3_ and NO_2_ on emergency room visits for respiratory diseases. **A** Effect of O_3_ under different NO_2_ levels; (**B**) Effect of NO_2_ under different O_3_ levels. NO_2_ or O_3_ concentrations were classified as low, medium, and high levels according to their 25% and 75% quartiles. NOTE: O_3_, ozone; NO_2_, nitrogen dioxide; TRD, total respiratory disease; AURI, acute upper respiratory infection; LRTI, lower respiratory tract infection

**Fig. 4:**
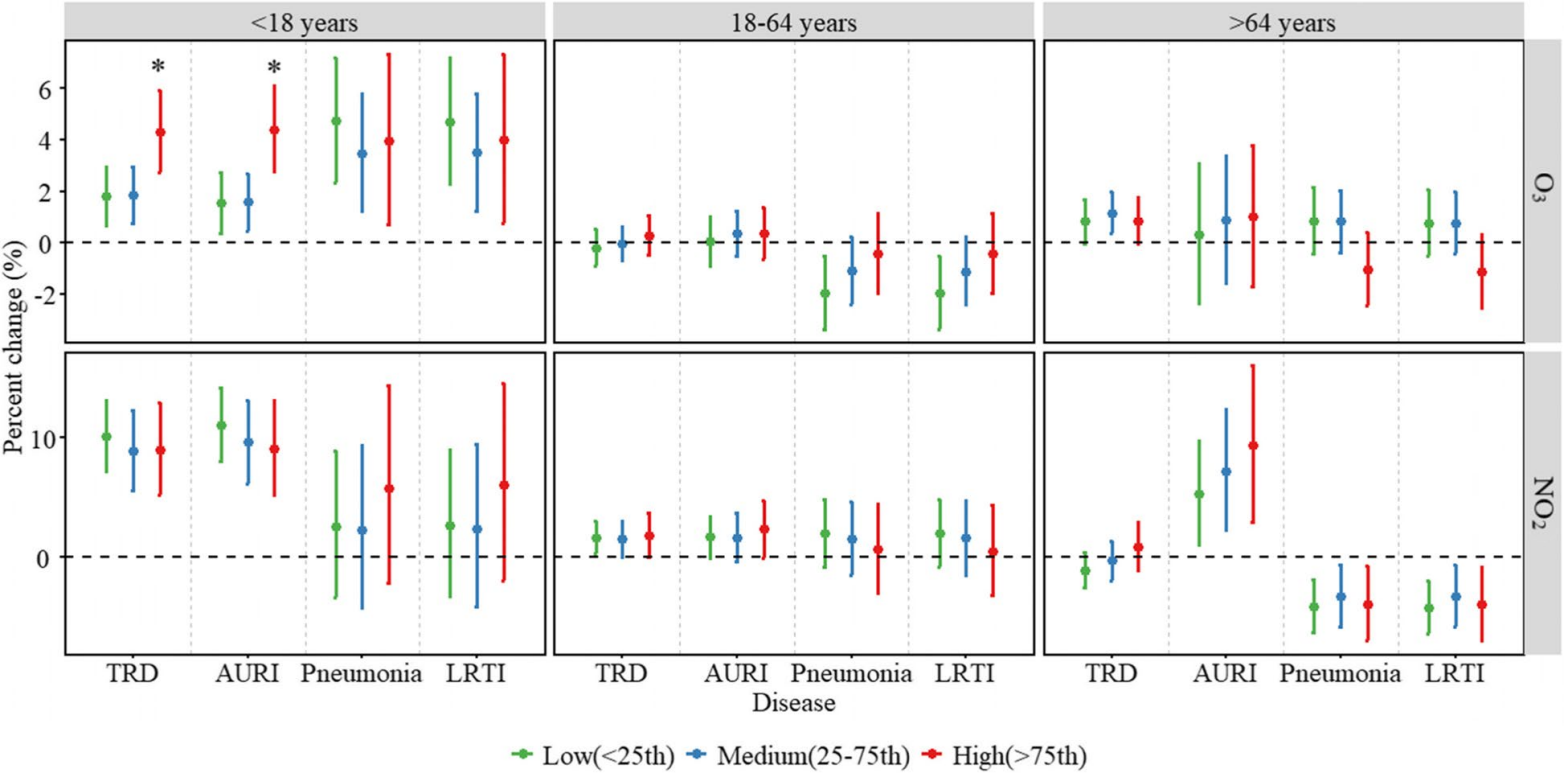
The interaction of O_3_ and NO_2_ on emergency room visits for respiratory diseases in different age groups (< 18 years, 18–64 years and > 64 years). NO_2_ or O_3_ concentrations were classified as low, medium, and high levels according to their 25% and 75% quartiles. NOTE: O_3_, ozone; NO_2_, nitrogen dioxide; TRD, total respiratory disease; AURI, acute upper respiratory infection; LRTI, lower respiratory tract infection..^*^P for interaction < 0.05

The results of interaction analysis between O_3_ and NO_2_ were shown in Fig. 3. We found an increasing trend in the associations between O_3_ and total visits and acute upper respiratory infection with increasing NO_2_ concentrations, although no significant differences were observed. However, no similar trend was observed for NO_2_. The interaction between O_3_ and NO_2_ was further analyzed in various subgroups of the population. Similar results were observed in both gender groups (Figure S6). Figure 4 showed the results of interaction analysis between O_3_ and NO_2_ in different age groups. The association between O_3_ and total respiratory emergency room visits and acute upper respiratory infection was significantly stronger at high concentrations of NO_2_ in the < 18 years group (*P*-interaction < 0.05).

### Sensitive analyses

Sensitive analyses indicated that, when additionally adjusting for co-pollutants by constructing a two-pollutant model, applying different lagged patterns for temperature, and using different degrees of freedom for calendar time, the associations between air pollutants and respiratory emergency room visits were generally robust, as shown in Table S1.

## Discussion

Air pollution is one of the important public health problems that threaten the health of people all over the world. Previous epidemiological studies demonstrated that air pollution is associated with respiratory diseases and other adverse health effects [31, 32]. In China and many other regions around the world, the adverse health effects of O_3_ and traffic-related pollution (e.g., NO_2_) on the respiratory system of populations are receiving increasing attention. The present study investigated the associations of short-term exposure to O_3_ and NO_2_ with ERVs for respiratory diseases based on 144,326 cases from a large general hospital in Beijing. We observed that short-term exposure to air pollutants (O_3_ and NO_2_) was positively associated with respiratory emergency room visits, particularly in acute upper respiratory infection and younger people (< 18 years). The adverse health effect of O_3_ was significantly strengthened at high NO_2_ concentration levels in the < 18 years group.

Previous studies have investigated associations between air pollution and respiratory ERVs. A study was performed in Colombia and the results showed a stronger association between NO_2_ concentration and the percentage increases in ERVs for respiratory diseases, especially in the 5 to 9-year-old age group [33]. An association was also found between O_3_ concentration and increased visits for respiratory diseases in children less than 5 years of age. Szyszkowicz M et al. [5] investigated associations between ambient air pollutants (PM_2.5_, NO_2_, O_3_ and SO_2_) and ERVs for respiratory diseases stratified by sex in Canada, and found that short-term exposure to air pollution increased the risk of ERVs for upper and lower respiratory diseases among males and females. In Chengdu, China, another study suggested that for respiratory disease visits, males were affected by the combination of PM_2.5_ and O_3_, but females were affected by PM_2.5_ only [9]. Previous studies have generally shown that short-term exposure to O_3_ and NO_2_ is positively associated with respiratory emergency room visits, but the results vary by cities and the sensitivity to pollutants varies by age and gender. In this study, we observed positive associations of O_3_ and NO_2_ with respiratory emergency room visits, especially in acute upper respiratory infection, which provided new evidence for the short-term effects of O_3_ and NO_2_ on the human respiratory system. There are several plausible mechanisms and pathways through which air pollutants could affect the respiratory system, including direct airway irritation causing bronchoconstriction, and oxidative stress with inflammation. The reactive oxygen species (ROS) generation of air pollutants can lead to oxidative injuries and systematic inflammatory responses, such as the generation of superoxide radical [34]. Cellular level stress and inflammation may also predispose the individual to subsequent infection and allergic sensitization [35]. O_3_ and NO_2_ are the two main oxidative gaseous pollutants in the atmosphere, and can cause systematic oxidative injuries and airway inflammatory [15, 36–38]. A few previous literatures [8, 34, 39] have reported that O_x_ and O_x_^wt^ were significantly correlated with fractional exhaled nitric oxide (FeNO), which was a biomarker of airway inflammation and could be useful for assessing the respiratory adverse effects of short-term air pollution exposure. Futhermore, O_x_^wt^ should be considered as a proxy indicator of NO_2_ and O_3_. The observation was consistent with our finding that O_X_ and O_x_^wt^ showed significantly positive correlations with ERVs for respiratory diseases over the entire concentration range. Future studies are needed to assess whether O_x_ and O_x_^wt^ can be used as a better indicator to estimate the effects of oxidative gaseous pollutants on respiratory diseases than O_3_ and NO_2_. There are few studies focused on the relationship between gas pollutants and ERVs for pneumonia. A meta-analysis including 21 studies showed significantly positive association between NO_2_ and hospital admission or ERVs for pneumonia, although no such correlation was identified regarding O_3_ [40]. In our study, there was no significant relationship between air pollutants (NO_2_, O_3_, O_x_ and O_x_^wt^) and pneumonia, which is not entirely consistent with the previous findings. The gaseous pollutants caused damage to the cells of the respiratory tract by impairing the membrane structures of the cell, pump structures within the cell membrane, and energy system, thus increasing the risk of infection [41]. However, these effects may depend on the levels of gaseous pollutants in lower respiratory tract, incubation period after exposure, susceptibility of population, and mixed effects between air pollutants. More high-quality studies are needed in the future to confirm the relationship between gaseous pollutants and ERVs for lower respiratory tract infection.

In gender-stratified analysis, we found that females seem to be more susceptible to ambient air pollution than males, which is consistent with the results of previous studies [31, 42–44]. Hormones and structural/morphological differences in the respiratory system may affect the differences in risks of air pollution exposure between men and women [45]. Compared with males, females have smaller respiratory tract, so they would be subjected to greater airway reactivity under the same air pollution [32, 46]. In age-stratified analysis, for respiratory diseases, the influence of air pollution seemed to be more obvious in children aged < 18 years group and aged > 65 years than those aged 18–64 years, which might be explained by the fact that the children and the elderly are susceptible groups due to weaker resistance to diseases [32]. This similar results have been seen in many other studies as well. In Sichuan, China, children (≤ 14 years) and elderly (≥ 65 years) appeared to be more vulnerable to the effects of air pollutants including PM_2.5,_ PM_10_, NO_2_, and SO_2_ [20]. Rodríguez-Villamizar LA et al. found that the effects of air pollutants on visits for respiratory diseases were greater for the 5 to 9-year-old group [33]. Children have higher breathing rates and are more likely to be outdoors, which results in higher exposure and inhalation of pollutants. In addition, children are more sensitive to pollutant stimulation due to underdeveloped lungs and smaller airway. In this study, we found a significant association between respiratory ERVs and O_3_ during warm season. On the contrary, the effect of NO_2_ on AURI was stronger during cold season. As a secondary air pollutant, surface O_3_ has higher concentrations in summer attributed to the more intensive sunlight and higher temperature, which favor the photochemical production of O_3_ [47]. In Beijing, the concentration of NO_2_ increased during the cold season due to the increased emissions of pollutants caused by winter heating. Meanwhile, low temperature would reduce the ability of the respiratory system to resist infection, which is related to the decrease of cilia clearance ability of the respiratory system and leukocyte phagocytosis [48]. These may be the reasons why NO_2_ caused stronger effects in the cold season.

Air pollution usually exists as a complex mixture and different pollutants may have potential interactions, therefore, it provides limited information to simply evaluate the health risk of a single pollutant. Thus, it is important to understand these possible potential interactions and synergy among air pollutants in order to evaluate the overall health effects of air pollution [49]. In this study, the effects on the association between O_3_ exposure and ERVs for respiratory diseases were stronger at high concentrations of NO_2_, particularly in younger people (< 18 years). The interaction mechanisms between O_3_ and NO_2_ on respiratory health effects are not entirely clear. First of all, through a series of complex photochemical reactions, a dynamic equilibrium is formed between NO, O_3_, and NO_2_ [50]. Furthermore, both O_3_ and NO_2_ are oxidative gaseous pollutants. O_3_ has a much stronger oxidation potential than NO_2_ and may have significant adverse effects even at low concentrations. Previous findings showed that low concentrations of O_3_ are associated with adverse cardiovascular outcomes in children, demonstrating that low concentrations of O_3_ can still have adverse effects in humans and that younger people may be a sensitive population. In addition, indoor O_3_ was found to pose a stronger adverse effect on heart rate variability in children at high levels of BC [18], which is consistent with our findings and implies a potential interaction between O_3_ and traffic-related pollutants on cardiopulmonary health of children. Excessive inhalation of O_3_ and NO_2_ can lead to imbalance of oxidation and anti-oxidation, thus causing oxidative stress and activating the release of inflammatory cytokines, resulting in oxidative stress damage and inflammatory response of respiratory epithelial cells. Moreover, synergistic effects of NO_2_ and O_3_ may also produce cumulative oxidative stress and thus causes more severe adverse respiratory effects [51, 52]. Generally speaking, the interactions and synergistic effects of O_3_ and NO_2_ cause more damage to respiratory system, but the mechanisms by which this damage occurs are still not fully understood. However, no similar trend was observed for NO_2_. The reason may be partially explained that NO_2_ concentration decreases (as Figure S7 showed) at high O_3_ concentration and thus the adverse respiratory effect is weaker.

The strength of this study is that we provide evidence of associations between gaseous air pollutants and emergency respiratory diseases in China. First, emergency department data have unique advantages in reflecting the acute effects after short-term air pollution exposure. Second, emergency department data can greatly avoid the interference of cross-regional visits (such as hospitalization data), which brings the advantage of exposure assessment. Third, this study provides the first evidence on the association of O_x_ and O_x_^wt^ with respiratory emergencies and interactions between O_3_ and NO_2_. However, our study still has some limitations. First, the data of ERVs were obtained from a single center within a limited area. Second, this is an ecological study that does not elucidate causality, and the findings and potential biological mechanisms still need to be confirmed by further studies. Third, the use of air pollutant data from environmental monitoring stations also has certain exposure bias, which is an inevitable limitation of ecological studies.

## Conclusions

Short-term exposure to O_3_ and NO_2_ was associated with increased emergency room visits for respiratory diseases, particularly acute upper respiratory infection. Meanwhile, younger people (< 18 years) were more sensitive to O_3_ and NO_2_, and the health risk of O_3_ is significantly enhanced at high NO_2_ concentration levels. Our findings provides new evidence for the development of targeted environmental health policies for specific diseases and populations.

## Supplementary Information

**Additional file 1.**

## Data Availability

The datasets generated and/or analysed during the current study are not publicly available but are available from the corresponding author on reasonable request.

## Abbreviations

O3: Ozone
NO2: Nitrogen dioxide
ERVs: Emergency room visits
Ox: Combined oxidant capacity
Oxwt: Redox-weighted oxidant capacity
TRDs,: Total respiratory diseases
AURI: Acute upper respiratory infection
EMS: Emergency medical service
COPD: Chronic obstructive pulmonary disease
PUTH: Peking University Third Hospital
ICD: International Classification of Disease
LRTI: Lower respiratory tract infection
PM2.5: Fine particles
PM10: Inhalable particles
BC: Black carbon
SO2: Sulfur dioxide
CO: Carbon monoxide
O3-8 h max: 8-h maximum concentration of O3
RH: Relative humidity
DLNM: Distributed lag nonlinear model
DOW: Day of Week
CI: Confidence interval;VIF, variance inflation factor
SD: Standard deviation
ROS: Reactive oxygen species
FeNO: Fractional exhaled nitric oxide
