# Supplementary material for: Association and interaction of O3 and NO2 with emergency room visits for respiratory diseases in Beijing, China: a time-series study
Source: BMC Public Health. 2022 Dec 5;22:2265. doi: 10.1186/s12889-022-14473-2 (PMC9721066; doi:10.1186/s12889-022-14473-2)
Supplement: Supplementary file 1 — Additional file 1. [file 12889_2022_14473_MOESM1_ESM.docx]

**Association and interaction of O_3_ and NO_2_ with emergency room visits for respiratory diseases in Beijing, China: A time-series study**

Yuanwei Fu^a,1^, Wenlou Zhang ^b,1^, Yan Li^a,1^, Hongyu Li^b^, Furong Deng ^b,^*, Qingbian Ma ^a,^*

^a^ Emergency Department, Peking University Third Hospital, Beijing 100191, China.

^b^ Department of Occupational and Environmental Health Sciences, School of Public Health, Peking University, Beijing 100191, China.

*Corresponding author: [maqingbian@bjmu.edu.cn](mailto:maqingbian@bjmu.edu.cn); frdeng@bjmu.edu.cn

^1^ These authors contributed equally to this work.

Table S1. The results of sensitive analysis

|  | O_3_ | NO_2_ | O_x_ | O_x_^wt^ |
| --- | --- | --- | --- | --- |
| +PM_2.5_ | | | | |
| TRD | 0.61 (0.01, 1.21) | 2.37 (0.57, 4.21) | 0.93 (0.31, 1.55) | 1.20 (0.26, 2.15) |
| AURI | 0.55 (-0.26, 1.37) | 4.53 (2.04, 7.07) | 1.11 (0.27, 1.95) | 1.30 (0.03, 2.58) |
| Pneumonia | 0.60 (-0.29, 1.51) | -2.81 (-5.4, -0.14) | 0.31 (-0.62, 1.23) | 0.71 (-0.69, 2.12) |
| LRTI | 0.56 (-0.33, 1.46) | -2.69 (-5.28, -0.03) | 0.28 (-0.64, 1.20) | 0.65 (-0.74, 2.06) |
| COPD | -0.01 (-2.76, 2.82) | 2.29 (-4.95, 10.08) | 0.36 (-2.5, 3.31) | 0.28 (-4.03, 4.78) |
| Asthma | 1.28 (-1.35, 3.99) | -1.35 (-8.88, 6.80) | 1.22 (-1.52, 4.04) | 1.96 (-2.17, 6.27) |
| +PM_10_ | | | | |
| TRD | 0.66 (0.07, 1.25) | 2.31 (0.64, 4.01) | 0.93 (0.35, 1.52) | 1.26 (0.35, 2.17) |
| AURI | 0.71 (-0.09, 1.51) | 4.85 (2.53, 7.23) | 1.27 (0.48, 2.06) | 1.57 (0.35, 2.81) |
| Pneumonia | 0.48 (-0.40, 1.37) | -3.81 (-6.18, -1.38) | -0.01 (-0.88, 0.88) | 0.36 (-1.00, 1.73) |
| LRTI | 0.44 (-0.44, 1.33) | -3.72 (-6.09, -1.29) | -0.04 (-0.91, 0.84) | 0.30 (-1.05, 1.67) |
| COPD | 0.20 (-2.52, 3.00) | 3.22 (-3.67, 10.60) | 0.72 (-2.01, 3.51) | 0.74 (-3.45, 5.12) |
| Asthma | 1.21 (-1.39, 3.88) | -1.14 (-8.39, 6.69) | 1.10 (-1.52, 3.80) | 1.81 (-2.21, 5.99) |
| +SO_2_ | | | | |
| TRD | 0.82 (0.24, 1.40) | 2.77 (1.32, 4.25) | 1.07 (0.53, 1.60) | 1.51 (0.65, 2.37) |
| AURI | 0.97 (0.18, 1.76) | 5.01 (2.95, 7.12) | 1.46 (0.74, 2.19) | 1.96 (0.79, 3.14) |
| Pneumonia | 0.56 (-0.31, 1.43) | -1.17 (-3.22, 0.93) | 0.30 (-0.50, 1.09) | 0.65 (-0.63, 1.94) |
| LRTI | 0.51 (-0.35, 1.38) | -1.16 (-3.20, 0.93) | 0.26 (-0.53, 1.05) | 0.58 (-0.69, 1.87) |
| COPD | 0.64 (-2.05, 3.40) | 4.27 (-1.39, 10.25) | 1.32 (-1.11, 3.81) | 1.64 (-2.32, 5.76) |
| Asthma | 0.88 (-1.62, 3.44) | -0.73 (-6.95, 5.91) | 0.66 (-1.64, 3.00) | 1.19 (-2.48, 5.00) |
| +CO | | | | |
| TRD | 0.77 (0.19, 1.35) | 2.61 (0.72, 4.53) | 0.93 (0.38, 1.49) | 1.33 (0.46, 2.21) |
| AURI | 0.83 (0.05, 1.61) | 3.68 (1.09, 6.32) | 1.09 (0.33, 1.85) | 1.50 (0.32, 2.69) |
| Pneumonia | 0.58 (-0.29, 1.46) | -1.45 (-4.21, 1.39) | 0.41 (-0.43, 1.26) | 0.76 (-0.54, 2.09) |
| LRTI | 0.53 (-0.33, 1.41) | -1.37 (-4.13, 1.46) | 0.38 (-0.46, 1.22) | 0.70 (-0.61, 2.02) |
| COPD | 0.62 (-2.07, 3.38) | 5.51 (-2.57, 14.25) | 1.12 (-1.44, 3.74) | 1.39 (-2.62, 5.57) |
| Asthma | 0.82 (-1.68, 3.39) | -3.91 (-11.54, 4.38) | 0.41 (-1.98, 2.86) | 0.93 (-2.80, 4.80) |
| 30-day lag for temperature | | | | |
| TRD | 1.01 (0.47, 1.57) | 3.21 (1.97, 4.47) | 1.33 (0.83, 1.82) | 1.90 (1.09, 2.71) |
| AURI | 1.06 (0.32, 1.81) | 5.52 (3.75, 7.32) | 1.69 (1.02, 2.36) | 2.25 (1.16, 3.35) |
| Pneumonia | 1.34 (0.50, 2.19) | -1.22 (-3.01, 0.61) | 0.86 (0.11, 1.62) | 1.70 (0.47, 2.95) |
| LRTI | 1.29 (0.46, 2.14) | -1.18 (-2.97, 0.64) | 0.83 (0.09, 1.58) | 1.64 (0.42, 2.88) |
| COPD | 0.89 (-1.71, 3.56) | 7.60 (2.70, 12.73) | 2.40 (0.12, 4.74) | 2.90 (-0.92, 6.86) |
| Asthma | 1.63 (-0.81, 4.12) | 0.11 (-5.18, 5.69) | 1.31 (-0.85, 3.52) | 2.30 (-1.24, 5.97) |
| Df of calendar time = 4 | | | | |
| TRD | 0.38 (-0.21, 0.97) | 3.15 (1.89, 4.42) | 0.86 (0.33, 1.39) | 1.04 (0.18, 1.91) |
| AURI | 0.35 (-0.44, 1.15) | 5.61 (3.80, 7.44) | 1.21 (0.49, 1.93) | 1.33 (0.17, 2.52) |
| Pneumonia | 0.52 (-0.34, 1.39) | 0.30 (-1.48, 2.1) | 0.47 (-0.30, 1.24) | 0.78 (-0.48, 2.06) |
| LRTI | 0.46 (-0.40, 1.32) | 0.28 (-1.49, 2.08) | 0.42 (-0.35, 1.19) | 0.70 (-0.56, 1.97) |
| COPD | 0.31 (-2.25, 2.95) | 5.75 (1.18, 10.53) | 1.68 (-0.57, 3.98) | 1.83 (-1.93, 5.73) |
| Asthma | 0.90 (-1.49, 3.35) | -2.48 (-7.35, 2.64) | 0.28 (-1.84, 2.44) | 0.87 (-2.59, 4.45) |
| Df of calendar time = 10 | | | | |
| TRD | 0.48 (-0.10, 1.06) | 3.22 (1.96, 4.50) | 0.93 (0.42, 1.45) | 1.18 (0.33, 2.03) |
| AURI | 0.30 (-0.48, 1.08) | 5.32 (3.54, 7.13) | 1.10 (0.40, 1.81) | 1.20 (0.05, 2.36) |
| Pneumonia | 0.64 (-0.22, 1.51) | -0.34 (-2.16, 1.50) | 0.46 (-0.32, 1.24) | 0.85 (-0.41, 2.13) |
| LRTI | 0.57 (-0.29, 1.44) | -0.33 (-2.14, 1.51) | 0.40 (-0.37, 1.18) | 0.76 (-0.50, 2.04) |
| COPD | 1.04 (-1.71, 3.87) | 8.86 (3.69, 14.30) | 2.88 (0.44, 5.37) | 3.45 (-0.61, 7.68) |
| Asthma | 1.53 (-1.01, 4.14) | -0.72 (-6.09, 4.96) | 1.10 (-1.16, 3.41) | 2.05 (-1.66, 5.89) |

NOTE: PM_2.5_, fine particles; PM_10_, inhalable particles; NO_2_, nitrogen dioxide; O_3_, ozone; SO_2_, sulfur dioxide; CO, carbon monoxide; O_X_, oxidant capacity; O_x_^wt^, redox-weighted oxidant capacity; TRD, total respiratory disease; AURI, acute upper respiratory infection; LRTI, lower respiratory tract infection; COPD, chronic obstructive pulmonary disease; DF, degree of freedom.


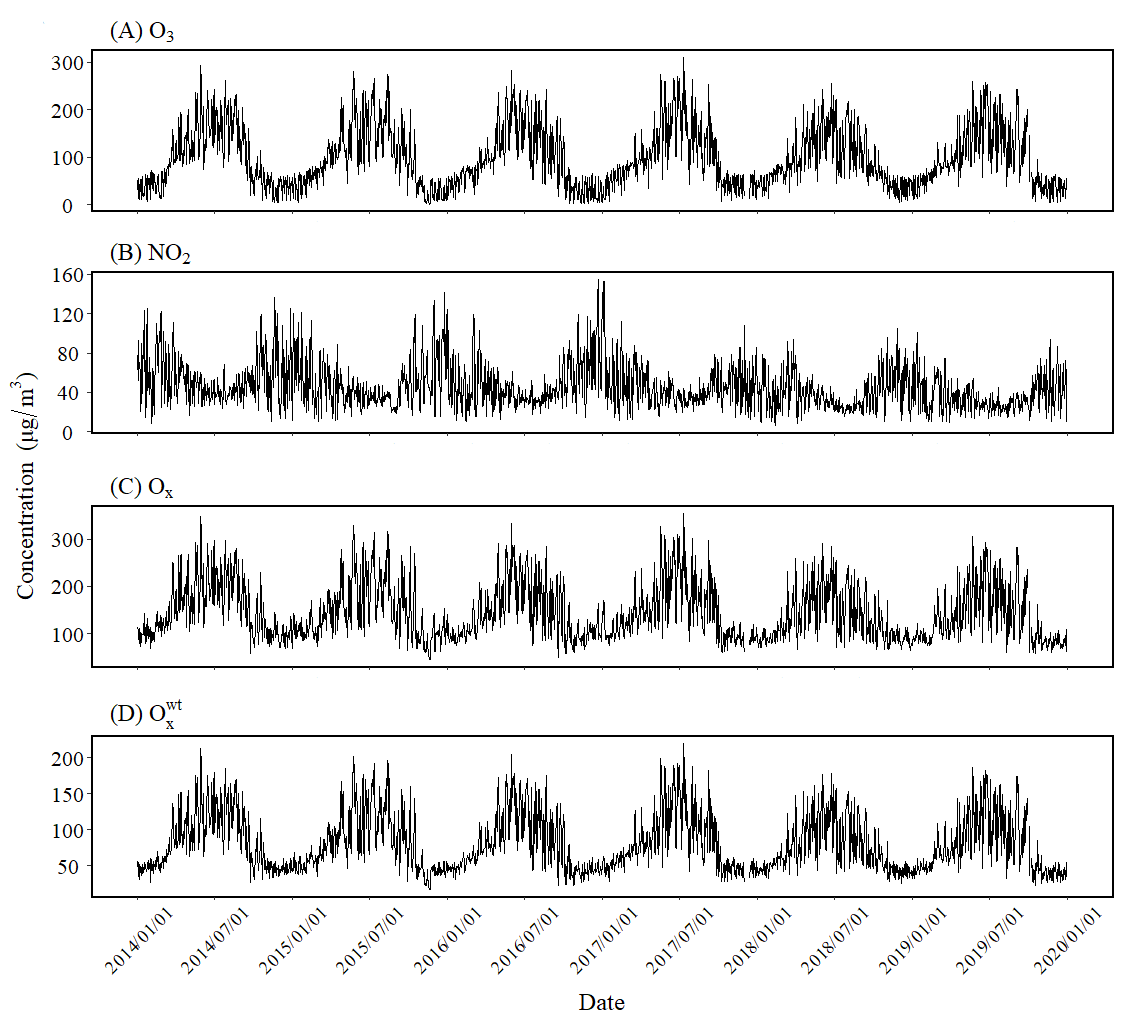


Figure S1. Time-series plot of the concentration of air pollutants (O_3_, NO_2_, O_X_ and O_x_^wt^) from Jan 1, 2014 to Dec 31, 2019. Notes: O_3_, ozone; NO_2_, nitrogen dioxide; O_X_, oxidant capacity; O_x_^wt^, redox-weighted oxidant capacity.


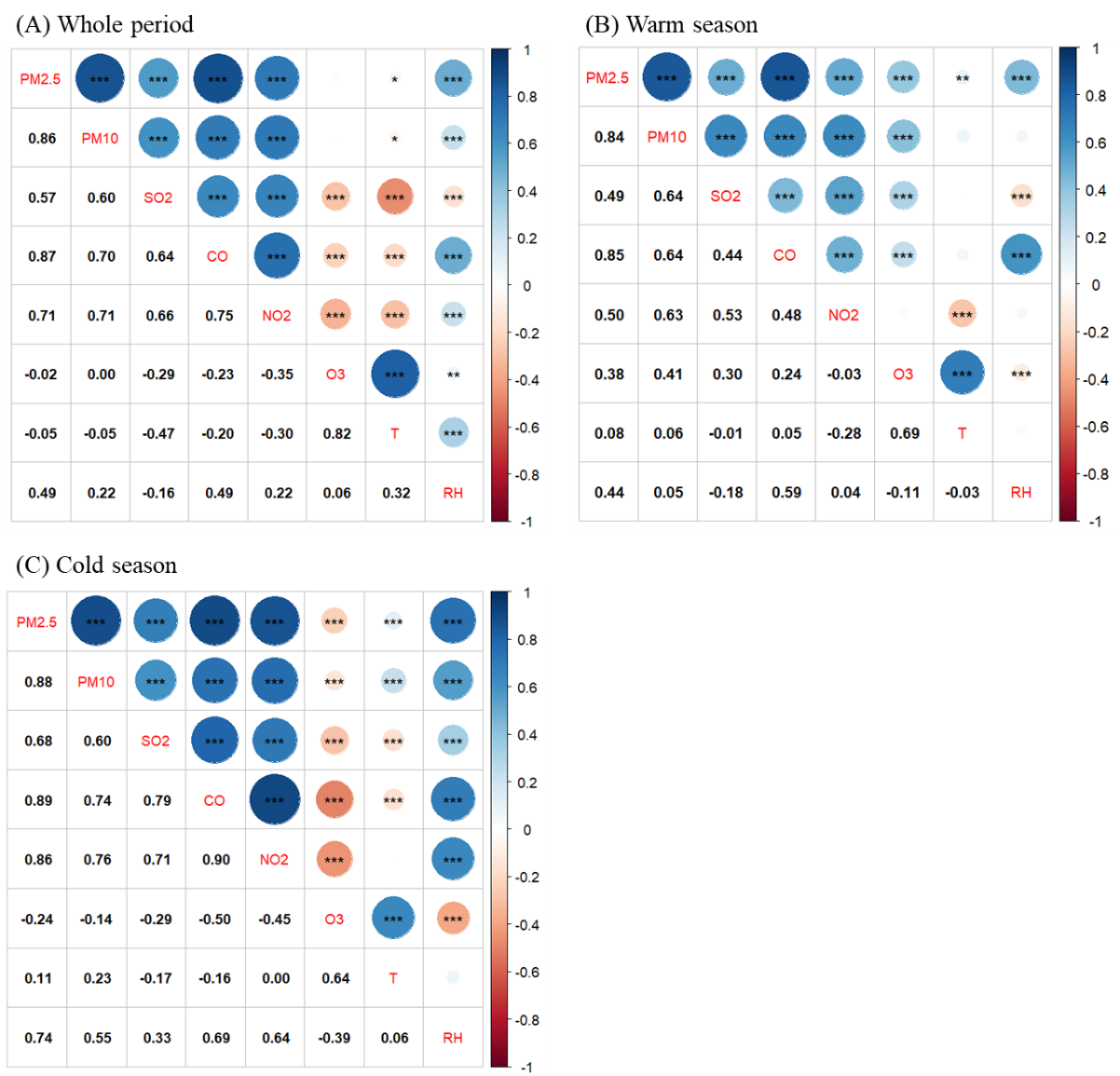


Figure S2. Spearman correlation coefficients between air pollutants and meteorological factor during the whole study period (A), warm season (B) and cold season (C).

^*^ *P* <0.05; ^**^ *P* < 0.01; ^***^ *P* < 0.001.


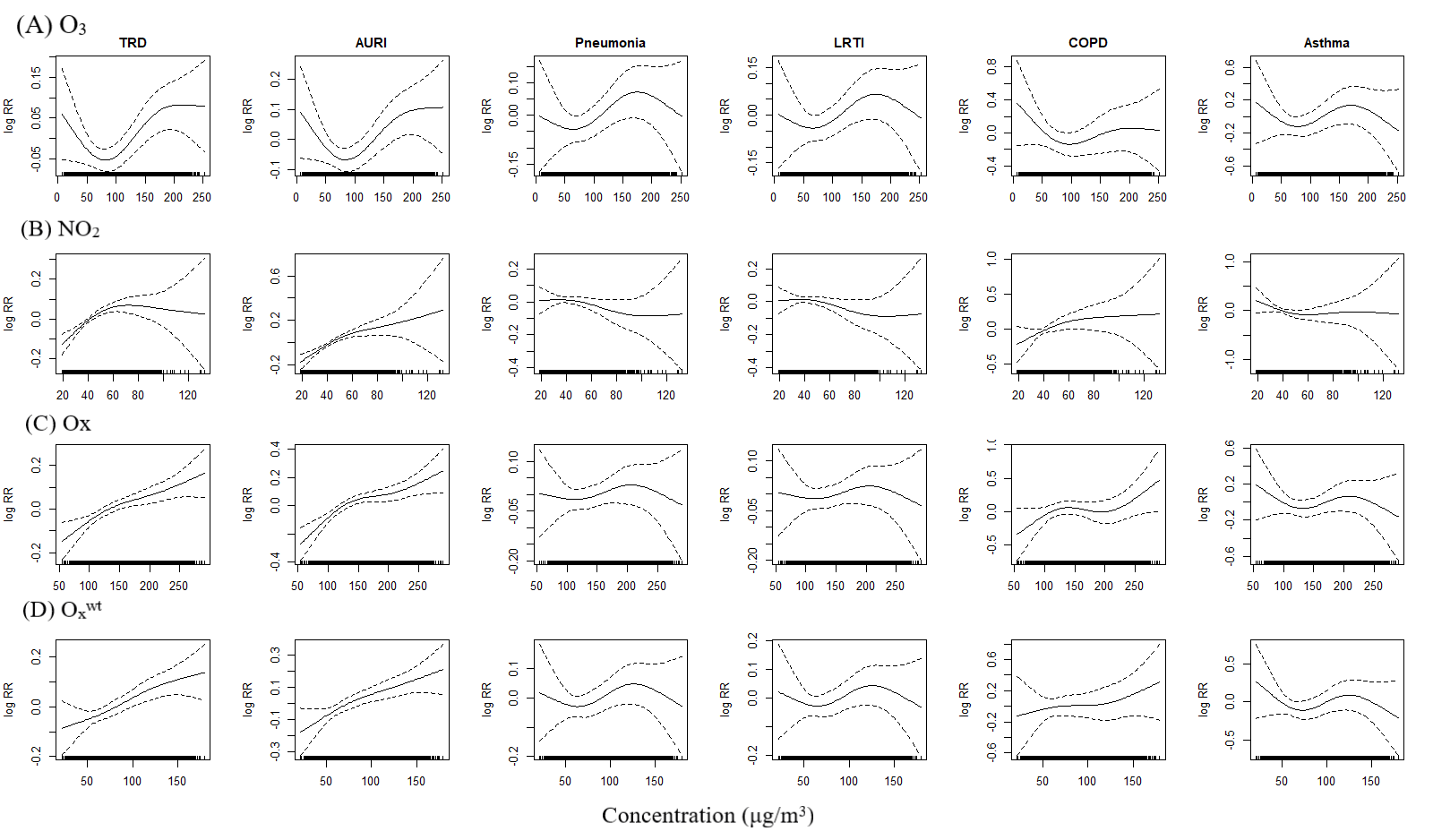


Figure S3. The exposure-response relationship curves between air pollutants and daily emergency room visits for respiratory diseases. NOTE: O_3,_ ozone; NO_2_, nitrogen dioxide; O_X_, oxidant capacity; O_x_^wt^, redox-weighted oxidant capacity; TRD, total respiratory disease; AURI, acute upper respiratory infection; LRTI, lower respiratory tract infection; COPD, chronic obstructive pulmonary disease.


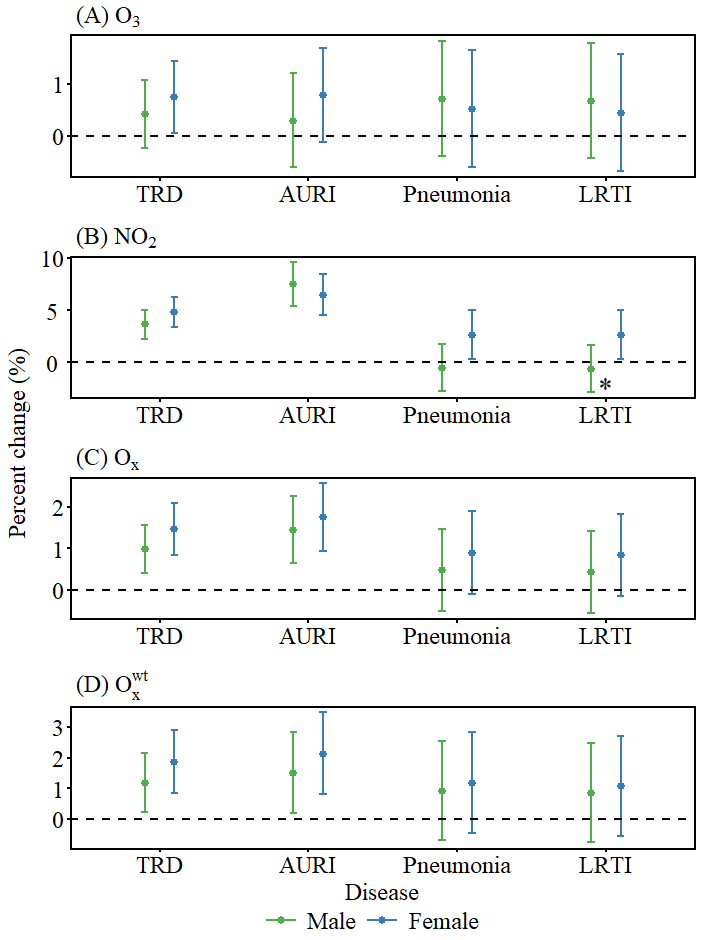


Figure S4. Associations of air pollutants (O_3_, NO_2_, O_X_ and O_x_^wt^) with emergency room visits for respiratory diseases stratified by gender (male and female) at lag 05 day during 2014-2019. NOTE: O_3_, ozone; NO_2_, nitrogen dioxide; O_X_, oxidant capacity; O_x_^wt^, redox-weighted oxidant capacity; TRD, total respiratory disease; AURI, acute upper respiratory infection; LRTI, lower respiratory tract infection. ^*^ *P* for subgroup differences <0.05.


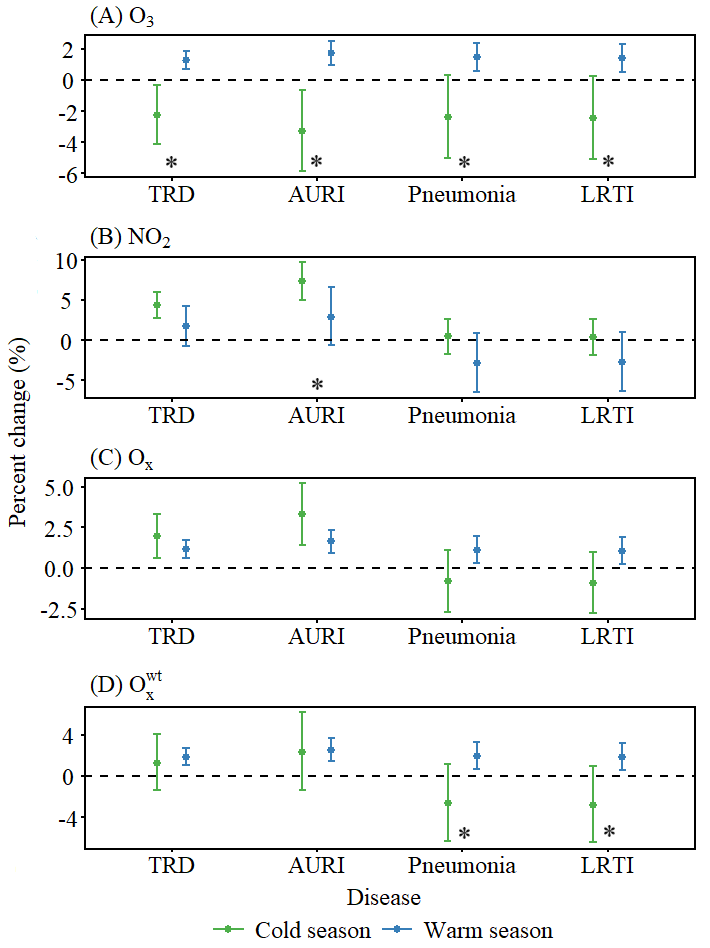


Figure S5. Associations of air pollutants (O_3_, NO_2_, O_X_ and O_x_^wt^) with emergency room visits for respiratory diseases stratified by season (warm: May to October and cool: November to April) at lag 05 day during 2014-2019. NOTE: O_3_, ozone; NO_2_, nitrogen dioxide; O_X_, oxidant capacity; O_x_^wt^, redox-weighted oxidant capacity; TRD, total respiratory disease; AURI, acute upper respiratory infection; LRTI, lower respiratory tract infection. ^*^ *P* for subgroup differences <0.05.


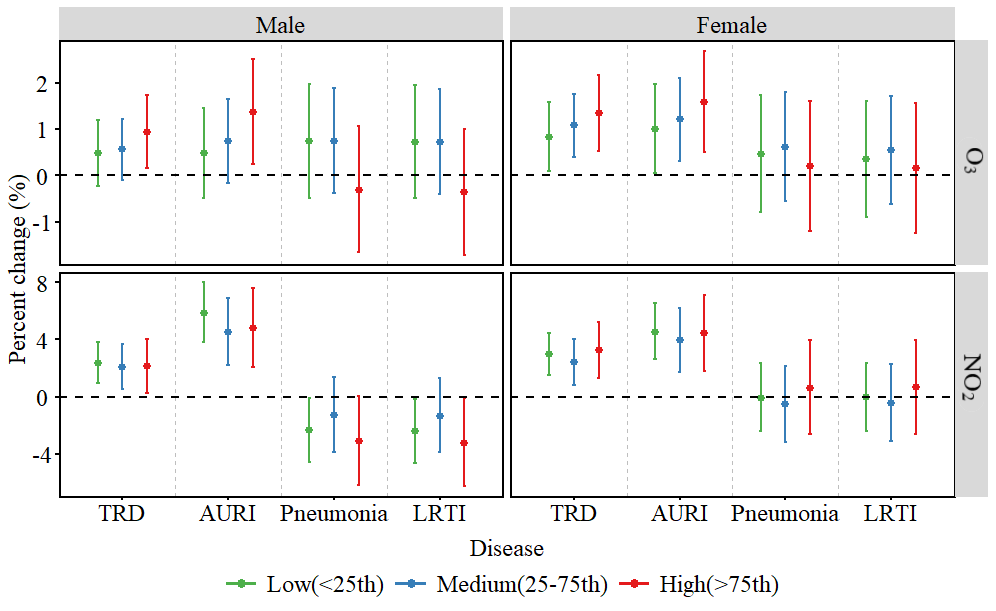


Figure S6. The interaction of O_3_ and NO_2_ on emergency room visits for respiratory diseases in different gender groups (male and female). NO_2_ or O_3_ concentration was classified as low, medium, and high levels according to their 25% and 75% quartiles. NOTE: O_3_, ozone; NO_2_, nitrogen dioxide; TRD, total respiratory disease; AURI, acute upper respiratory infection; LRTI, lower respiratory tract infection.


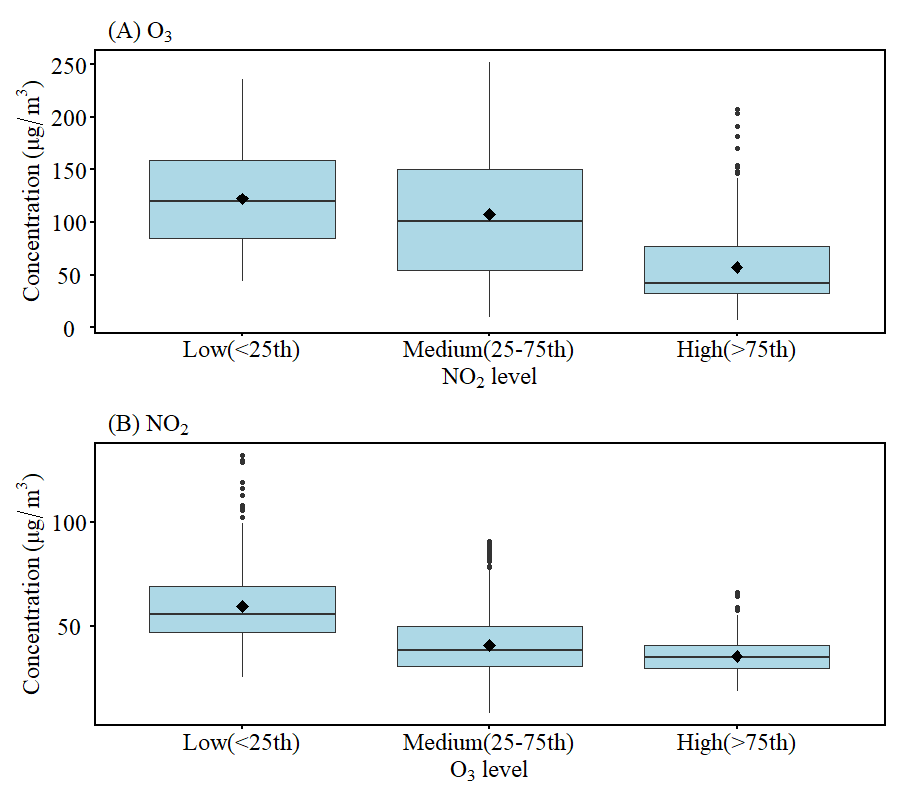


Figure S7. The relationship of concentration distribution between O_3_ and NO_2_ in the atmosphere. NO_2_ or O_3_ concentration was classified as low, medium, and high levels according to their 25% and 75% quartiles. NOTE: O_3_, ozone; NO_2_, nitrogen dioxide.
